# Comparison of Mortality Among Participants of Women’s Health Initiative Trials With Screening-Detected Breast Cancers vs Interval Breast Cancers

**DOI:** 10.1001/jamanetworkopen.2020.7227

**Published:** 2020-06-30

**Authors:** Veronica L. Irvin, Zhenzhen Zhang, Michael S. Simon, Rowan T. Chlebowski, Shiuh-Wen Luoh, Aladdin H. Shadyab, Jessica L. Krok-Schoen, Fred K. Tabung, Lihong Qi, Marcia L. Stefanick, Pepper Schedin, Sonali Jindal

**Affiliations:** 1 College of Public Health and Human Sciences, Oregon State University, Corvallis; 2 Division of Oncological Sciences, Oregon Health & Science University, Portland; 3 Knight Cancer Institute, Oregon Health & Science University, Portland; 4 Karmanos Cancer Institute, Department of Oncology, Wayne State University, Detroit, Michigan; 5 Lundquist Institute for Biomedical Innovation at Harbor-UCLA Medical Center, Torrance, California; 6 Department of Family Medicine and Public Health, School of Medicine, University of California, San Diego, La Jolla; 7 School of Health and Rehabilitation Sciences, The Ohio State University, Columbus; 8 College of Medicine and Comprehensive Cancer Center, The Ohio State University, Columbus; 9 Department of Public Health Sciences, University of California Davis School of Medicine, Davis; 10 Department of Medicine (Stanford Prevention Research Center), School of Medicine, Stanford University, Stanford, California; 11 Department of Cell, Developmental and Cancer Biology, Oregon Health & Science University, Portland

## Abstract

**Importance:**

Interval breast cancers (IBCs) are cancers that emerge after a mammogram with negative results but before the patient’s next scheduled screening. Interval breast cancer has a worse prognosis than cancers detected by screening; however, it is unknown whether the length of the interscreening period is associated with prognostic features and mortality.

**Objective:**

To compare the prognostic features and mortality rate of women with IBCs diagnosed within 1 year or between 1 and 2.5 years of a mammogram with negative results with the prognostic features and mortality rate of women with breast cancers detected by screening.

**Design, Setting, and Participants:**

This cohort study used mammography data, tumor characteristics, and patient demographic data from the Women’s Health Initiative study, which recruited participants from 1993 to 1998 and followed up with participants for a median of 19 years. The present study sample for these analyses included women aged 50 to 79 years who participated in the Women’s Health Initiative study and includes data collected through March 31, 2018. There were 5455 incidents of breast cancer; only 3019 women compliant with screening were retained in analyses. Statistical analysis was performed from October 25, 2018, to November 24, 2019. Breast cancers detected by screening and IBCs were defined based on mammogram history, date of last mammogram, type of visit, and results of examination. Interval breast cancers were subdivided into those occurring within 1 year or between 1 and 2.5 years after the last protocol-mandated mammogram with negative results.

**Main Outcomes and Measures:**

The primary outcome of this study was breast cancer–specific mortality for each case of breast cancer detected by screening and IBCs detected within 1 year or between 1 and 2.5 years from a mammogram with negative results. Secondary outcomes included prognostic and tumor characteristics for each group. Comparisons between groups were made using the *t* test, the χ^2^ test, and Fine-Gray multivariable cumulative incidence regression analyses.

**Results:**

Among the 3019 participants in this analysis, all were women with a mean (SD) age of 63.1 (6.8) years at enrollment and 68.5 (7.1) years at diagnosis. A total of 1050 cases of IBC were identified, with 324 (30.9%) diagnosed within 1 year from a mammogram with negative results and 726 (69.1%) diagnosed between 1 and 2.5 years after last mammogram with negative results. The remaining 1969 cases were breast cancers detected by screening. Interval breast cancers diagnosed within 1 year from a mammogram with negative results had significantly more lobular histologic characteristics (13.0% vs. 8.1%), a larger tumor size (1.97 cm vs 1.43 cm), a higher clinical stage (28.4% vs 17.3% regional and 3.7% vs 0.6% distant), and more lymph node involvement (27.1% vs 17.0%) than cancers detected by screening. Unadjusted breast cancer–specific mortality hazard ratios were significantly higher for IBCs diagnosed within 1 year from a mammogram with negative results compared with breast cancers detected by screening (hazard ratio, 1.92; 95% CI, 1.39-2.65). Higher breast cancer–specific mortality remained statistically significant for IBCs diagnosed within 1 year after adjusting for trial group, molecular subtype, waist to hip ratio, histologic characteristics, and either tumor size (hazard ratio, 1.46; 95% CI, 1.03-2.08) or lymph node involvement (hazard ratio, 1.44; 95% CI, 1.03-2.01). However, significance was lost when tumor size and lymph node involvement were both included in the model (hazard ratio, 1.34; 95% CI, 0.96-1.88). Interval breast cancers diagnosed between 1 and 2.5 years from a mammogram with negative results were not different from breast cancers detected by screening based on prognostic factors or mortality.

**Conclusions and Relevance:**

Women with IBCs diagnosed within 1 year of negative mammogram results overall were associated with worse survival than women with breast cancers detected by screening. These differences in survival may be due to a uniquely aggressive biology among IBC cases.

## Key Points

**Question:**

Is the length of the interscreening period associated with breast cancer prognostic factors and mortality in interval breast cancers compared with cancers detected by screening?

**Findings:**

In this study using data from the Women’s Health Initiative, a national study among postmenopausal women, interval breast cancers diagnosed within 1 year from a mammogram with negative results were associated with worse breast cancer–specific mortality compared with breast cancers detected by screening. Mortality remained statistically significantly higher after adjustment for trial group, molecular subtype, other risk factors, histologic characteristics, and either tumor size or lymph node but not when tumor size and lymph node were included in the model; no differences were observed between interval cancers diagnosed between 1 and 2.5 years from a mammogram with negative results and breast cancers detected by screening.

**Meaning:**

Interval cancers occurring within 1 year from a mammogram with negative results may have a unique biology that accounts for aggressive features.

## Teaser

This cohort study uses data from the Women’s Health Initiative study to compare the prognostic features and mortality rate of women with interval breast cancers diagnosed within 1 year or between 1 and 2.5 years of a mammogram with negative results with the prognostic features and mortality rate of women with breast cancers detected by screening.

## Introduction

Regular mammographic screening is associated with a more frequent diagnosis of early-stage cancer and an approximately 20% reduction in breast cancer mortality.^1,2,3^ However, screening mammograms miss 20% to 30% of breast cancers.^4^ Most of these missed cancers are interval breast cancers (IBCs), defined as cancers that emerge after nonsuspicious mammogram results but before the patient’s next scheduled screening. Interval breast cancer tumors are often diagnosed at a later stage than cancers detected by screening,^5,6,7,8^ and they are associated with a 2-fold to 3-fold increased risk of breast cancer–specific mortality compared with cancers detected by screening, even after adjustment for clinically relevant variables.^5,7,9,10^ Other studies report mortality rates among women with IBC that are comparable to those among women who did not undergo screening,^1,11^ emphasizing the need for additional study.

The rate and proportion of IBCs may vary depending on the age and breast cancer risk of the population, whether the test was an initial or repeated screening, and the length of the interscreening interval.^12^ The number of cases of IBCs is higher when the number of years between screenings is higher.^12^ The percentage of IBCs detected range between 12% and 26% for annual screenings, between 17% and 33% for biennial screenings, and between 32% and 38% for triennial screenings.^6,12,13,14,15^ Previous studies have not been able to determine whether the length of the interscreening interval accounts for variation in prognostic factors or mortality rates among women with IBCs. Using prospective cohort data from the Women’s Health Initiative (WHI), our study’s objective was to compare breast cancer prognostic factors and breast cancer–specific mortality between women with breast cancers detected by screening and women with IBCs diagnosed within 1 year or between 1 and 2.5 years after negative mammogram results.

## Methods

### Study Sample

This study is a secondary analysis of women from the WHI, a national cohort study of postmenopausal women, which has been previously described in detail.^16,17,18^ The study sample included women participating in the WHI clinical trials in either 1 of 2 hormone therapy (HT) trials, the dietary modification (DM) trial, or both an HT and DM trial. Women in the WHI observational study were not included because they did not undergo a protocol-mandated mammography and they had less detailed mammography intake information. Participants were enrolled at 40 US clinical centers between 1993 and 1998. Eligibility criteria were as follows: being between the ages of 50 and 79 years at entry, being postmenopausal with no previous breast cancer, and having entry mammogram results not suspicious for breast cancer. Mammography was protocol-mandated annually for women in an HT trial and biennually for women in the DM trial. The WHI protocol was approved by the institutional review boards at the Clinical Coordinating Center at the Fred Hutchinson Cancer Research Center in Seattle, Washington, and at each of the 40 clinical centers.^17^ The present study was approved by the institutional review boards at Oregon Health & Science University and Oregon State University. All patients provided written informed consent. This study followed the Strengthening the Reporting of Observational Studies in Epidemiology (STROBE) reporting guideline.

The present study sample included clinical trial participants who received a diagnosis of breast cancer during the intervention phase of the trial or during the subsequent follow-up period. Information on the mammogram results was collected for women in the DM group through 2005 and for women in the HT group through 2007. Subsequently, mammography was not protocol-mandated in the follow-up period, but women were encouraged to follow the screening frequency previously recommended and information on mammogram results continued to be collected. Women in both the DM and HT trials were followed up for mortality during the WHI extension studies, and the results reflect the findings through March 31, 2018. Further details on WHI study design, recruitment, and baseline characteristics have been previously reported.^17,18^

### IBC or Breast Cancer Detected by Screening Based on Mammogram Data Collection

The WHI protocol recommended that mammography be performed at an American College of Radiology–accredited or US Food and Drug Administration–accredited facility and that the mammograms be read by a qualified radiologist. The mammography intake form did not classify the breast cancers as being an interval cancer or as being detected by screening, but it did include a variable to differentiate mammograms for screening from mammograms for nonroutine reasons. Findings from the 2 to 3 mammograms prior to the breast cancer diagnosis were reviewed to discriminate between cancers detected by screening and IBCs. The mammogram form (form 85) was recorded in the WHI database, and a team composed of a breast cancer physician (S.W.L.), 2 epidemiologists (V.L.I. and Z.Z.), a breast cancer basic scientist (P.S.), and an MD-trained pathologist (S.J.) reviewed the historical information recorded in the database. The following variables were used: date of breast cancer diagnosis, date of mammogram, reason for visit (routine or nonroutine), repeated mammogram recommended, and the Breast Imaging Reporting and Data System categories (negative; benign finding [negative; probably benign finding] short-interval follow-up suggested; suspicious abnormality [biopsy should be considered]; and highly suggestive of malignant neoplasm). The length of the interscreening interval was defined as the time between the last negative mammogram result and the next recommended screening mammogram. Six months was added to the longest recommended screening period to allow time for women to keep their protocol-mandated screening appointment.

Breast cancer detected by screening was defined as a protocol-mandated mammogram during the intervention phase or a protocol-recommended mammogram screening that showed suspicious, highly suggestive, or probably benign findings with follow-up suggested and a diagnosis of cancer within 2.5 years.

Interval breast cancers were diagnosed within recommended screening intervals of 2.5 years for participants in the DM trial or 1.5 years for participants in the HT trial after either (1) a protocol-mandated or recommended screening with negative results or (2) a diagnostic mammogram that showed negative or probably benign findings with the second to the last protocol-mandated mammogram showing normal results. These 2 definitions were required because the WHI data set routinely captured data from protocol-mandated or protocol-recommended mammograms but did not always have record of a diagnostic mammogram. Interval breast cancers were subdivided to match the 2 recommended screening intervals—those diagnosed within 1 year of the last screening or those diagnosed between 1 and 2.5 years since the last screening (eFigure in the Supplement).

### Ascertainment of Breast Cancer, Tumor Characteristics, and Mortality

Medical history forms were completed semiannually during the intervention phase of the trials and annually during extension studies. Reported breast cancers were confirmed by medical record and pathology report reviewed initially by centrally trained physician adjudicators at the local clinical centers, with final verification and coding centrally at the WHI Clinical Coordinating Center. Tumor characteristics included lymph node involvement, tumor size, tumor stage, and histologic findings (coded as ductal, lobular, or other);whether the tumors tested positive for ERBB2 (formerly HER2 or HER2/neu), estrogen receptors, or progesterone receptors was determined by local laboratories.

Specific cause of death was determined by physician adjudicators or, in some cases, by relative report. Mortality information was enhanced by serial National Death Index queries (last conducted December 31, 2017), which capture 98% of US deaths.^19^ For this study, breast cancer–specific cause of death was the final primary outcome.

Data on demographic characteristics and common risk factors were collected at baseline, including trial assignment, HT use, age, race/ethnicity, income, educational level, height, weight, waist to hip ratio, family history, age at menarche and first birth, smoking, alcohol intake, and Charlson comorbidity index.^20^

### Statistical Analysis

Statistical analysis was performed from October 25, 2018, to November 24, 2019. We conducted χ^2^ tests for categorical variables and *t* tests for continuous variables when comparing demographic characteristics and prognostic factors by breast cancer group (ie, interval of <1 year, interval between 1 and 2.5 years, or breast cancer detected by screening). Logistic regression was used to compute an odds ratio for each of the 3 IBC outcomes (all IBC, IBC <1 year, or IBC 1-2.5 years) by the categorical variables (stage, grade, lymph node involvement, molecular type, and histologic type). Time between breast cancer diagnosis and death, loss to follow-up, or end of follow-up (whichever came first) was calculated. To account for competing risks of death, we used the unadjusted and adjusted Fine-Gray competing risks regression model for breast cancer–specific mortality. The primary outcome was breast cancer–specific mortality, the competing risk was any death due to reasons other than breast cancer (such as cardiovascular diseases), and the censoring variable was a variable indicating censored observations in which time to death was not observed for reasons such as loss to follow-up. Regression models were calculated by the sequential addition of demographic, clinical, and tumor characteristics. The variable selection was based on the results of the univariate analysis (eTable 1 in the Supplement). The following variables were included in the multivariable models: IBC or breast cancer detected by screening status, trial groups, molecular subtype, tumor histologic type, tumor size, lymph node involvement, total number of mammograms before diagnosis, age at diagnosis, and the variables listed under demographic and common risk factors. Stage was not included in the multivariable analyses because stage is a composite variable based on tumor size and lymph node involvement, which were included in the multivariable models. Model 1 included IBC and breast cancer detected by screening with molecular subtype, model 2 included histologic subtype, model 3 included HT clinical trial group and DM trial group, model 4 included waist to hip ratio, model 5 included tumor size, model 6 included lymph node involvement, and model 7 included age at diagnosis, race/ethnicity, family history of breast cancer, comorbidity, total number of mammograms before diagnosis, age at menarche, age at first birth, income, educational level, smoking, alcohol use, and body mass index (calculated as weight in kilograms divided by height in meters squared).

The following sensitivity analyses were conducted: (1) substitution of time in survival models from date of last mammogram through end of follow-up to control for lead-time bias, (2) replication of final model to include women not participating in the HT trials, and (3) replication of final model among women from just the placebo groups of both the DM and HT trials. All analyses were conducted using SAS, version 9.4 (SAS Institute Inc). Tests of statistical significance were determined using 2-sided tests, and *P* ≤ .05 was considered statistically significant.

## Results

**Figure 1.**
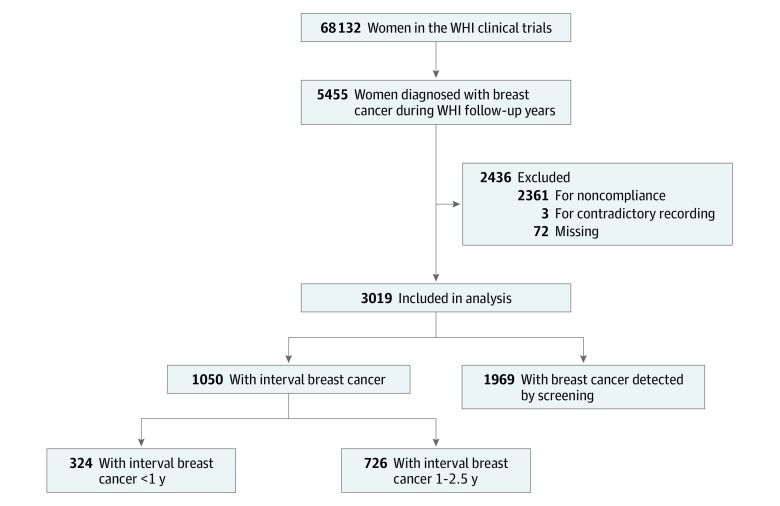
CONSORT Diagram of Women’s Health Initiative (WHI) Participants Included in the Analyses

A total of 5455 women with breast cancer were identified among 68 132 women during a median 19-year period, with 3019 women with breast cancer included in the analysis. The mean (SD) age at enrollment was 63.1 (6.8) years; the mean (SD) age at diagnosis was 68.5 (7.1) years (Table 1). A total of 2436 women were excluded for the following reasons: (1) did not follow protocol-mandated screening guidelines during their enrollment period because there was more than 2.5 years between the last mammogram with negative results and the diagnosis of breast cancer (n = 2361), (2) contradictory data (abnormal screening findings but final results recorded as normal) (n = 3), and (3) missing data (n = 72) (Figure 1). If women had 2 breast cancer diagnoses, data were retained from the initial cancer diagnosis (n = 85). Excluded women were significantly more likely than included women to be a participant of the DM trial (79.4%), to be younger at enrollment (mean [SD] age, 61.1 [6.4] years), and to be older at diagnosis (mean [SD] age, 75.4 [6.9] years). The mean (SD) time from enrollment to breast cancer diagnosis was 5.5 (3.0) years (median, 5 years; range, 0-22 years).

**Table 1.** Comparison of Baseline Demographic and Health Characteristics Between Women With a Diagnosis of Breast Cancer Detected by Screening vs Women With a Diagnosis of Interval Breast Cancer^a^

Characteristic	Breast cancer detected by screening (N = 1969)	All interval breast cancers (N = 1050)	*P* value^b^	Interval breast cancer <1 y (n = 324)^c^	*P* value^b^	Interval breast cancer 1-2.5 y (n = 726)^c^	*P* value^b^
Age at diagnosis, mean (SD), y	68.5 (7.1)	68.6 (7.3)	.97	68.1 (7.5)	.30	68.8 (7.3)	.49
BMI at enrollment, mean (SD)	29.5 (5.8)	28.6 (5.6)	<.001	28.1 (5.4)	<.001	28.8 (5.8)	.009
Waist to hip ratio at enrollment, mean (SD)	0.82 (0.08)	0.81 (0.08)	.003	0.81 (0.07)	.03	0.81 (0.08)	.01
Race/ethnicity, No. (%)							
White	1688/1965 (85.9)	910 (86.7)	.81	276 (85.2)	.24	634 (87.3)	.45
African American	160/1965 (8.1)	80 (7.6)		22 (6.8)		58 (8.0)	
Hispanic	44/1965 (2.2)	24 (2.3)		14 (4.3)		10 (1.4)	
Asian	45/1965 (2.3)	26 (2.5)		8 (2.5)		18 (2.5)	
Other	28/1965 (1.4)	10 (1.0)		4 (1.2)		6 (0.8)	
Missing	4	0		0		0	
Family history of breast cancer, No. (%)							
Yes	423/1864 (22.7)	255/1005 (25.4)	.11	80/309 (25.9)	.22	175/696 (25.1)	.19
No	1441/1864 (77.3)	750/1005 (74.6)		229/309 (74.1)		521/696 (74.9)	
Missing	105	45		15		30	
Ever full-term birth, No. (%)							
Yes	1732/1783 (97.1)	894/923 (96.9)	.68	273/283 (96.5)	.54	621/640 (97.0)	.89
No	51/1783 (2.9)	29/923 (3.1)		10/283 (3.5)		19/640 (3.0)	
Missing	186	127		41		86	
HT study group status^d^, No. (%)							
Estrogen-alone intervention	131/927 (14.1)	32/209 (15.3)	.25	20/131 (15.3)	.37	12/78 (15.4)	.70
Estrogen-alone control	191/927 (20.6)	34/209 (16.3)		21/131 (16.0)		13/78 (16.7)	
Estrogen plus progestin intervention	341/927 (36.8)	90/209 (43.1)		57/131 (43.5)		33/78 (42.3)	
Estrogen plus progestin control	264/927 (28.5)	53/209 (25.4)		33/131 (25.2)		20/78 (25.6)	
Not randomized to HT	1042	841		193		648	
DM trial group status^e^, No. (%)							
Intervention	521/1343 (38.8)	346/899 (38.5)	.88	92/232 (39.7)	.80	254/667 (38.1)	.76
Control	822/1343 (61.2)	553/899 (61.5)		140/232 (60.3)		413/667 (61.9)	
Not randomized to DM	626	151		92		59	
Comorbidity at enrollment, No. (%)							
0	1365/1969 (69.3)	741 (70.6)	.83	222 (68.5)	.45	519 (71.5)	.37
1	423/1969 (21.5)	212 (20.2)		66 (20.4)		146 (20.1)	
2	132/1969 (6.7)	73 (7.0)		23 (7.1)		50 (6.9)	
≥3	49/1969 (2.5)	24 (2.3)		13 (4.0)		11 (1.5)	

Abbreviations: BMI, body mass index (calculated as weight in kilograms divided by height in meters squared); DM, dietary modification; HT, hormone therapy.

^a^ Interval breast cancers were subdivided by their interscreening period: those diagnosed within 1 year of last screening or between 1 and 2.5 years since last screening.

^b^ The χ^2^ test was used for categorical variables, and the *t* test was used for continuous variables.

^c^ Missing categories are excluded from statistical analysis.

^d^ Calculated without including “not randomized to HT group.”

^e^ Calculated without including “not randomized to DM group.”

A total of 1969 cases of breast cancer (65.2%) were detected by screening, and 1050 (34.8%) were IBCs. Most cases of breast cancer detected by screening (1891 of 1969 [96.0%]) were diagnosed within 1 year after the mammogram. More than twice as many IBCs were diagnosed between 1 and 2.5 years after negative mammogram results (n = 726) compared with those diagnosed within 1 year of negative mammogram results (n = 324).

Interval breast cancers were compared with breast cancers detected by screening based on participant demographic characteristics and tumor characteristics. Women with IBCs had a significantly lower mean (SD) body mass index (28.6 [5.6] vs 29.5 [5.8]) and mean (SD) waist to hip ratio (0.81 [0.08] vs 0.82 [0.08]) than women with cancers detected by screening (Table 1). There were no differences by age at diagnosis, race/ethnicity, family history of breast cancer, parity, comorbidity, or trial group assignment between breast cancers detected by screening and IBCs regardless of the length of the interscreening period.

Table 2 shows the comparison of tumor characteristics between women who received a diagnosis of breast cancer detected by screening and women with an IBC by length of interscreening period.^21^ Tumor characteristics significantly differed between IBCs and breast cancers detected by screening, but only for IBCs diagnosed within 1 year of negative mammogram results. Compared with breast cancers detected by screening, IBCs diagnosed within 1 year of negative mammogram results had a significantly larger mean (SD) tumor size (1.97 [1.61] cm vs 1.43 [1.23] cm), were more likely to have tumors that were regional stage (91 of 321 [28.4%] vs 336 of 1948 [17.3]%) or distant stage tumors (12 of 321 [3.7%] vs 11 of 1948 [0.6%]), and were found to have higher proportions with lymph node involvement (83 of 306 [27.1%] vs 326 of 1919 [17.0%]) and lobular histologic characteristics (42 of 322 [13.0%] vs 159 of 1966 [8.1%]). No differences in tumor characteristics (such as size, grade, histologic characteristics, or lymph node involvement) were observed between IBCs diagnosed between 1 and 2.5 years and breast cancers detected by screening. Furthermore, no differences in molecular type (progesterone receptor, ERBB2, or triple negative) were observed between breast cancers detected by screening and IBCs diagnosed within either length of interscreening period.

**Table 2.** Comparison of Tumor Characteristics Between Women With a Diagnosis of Breast Cancer Detected by Screening vs Women Diagnosed With Interval Breast Cancer by Length of Interscreening Period^a^

Characteristic	Breast cancer detected by screening (N = 1969)	All interval breast cancers (N = 1050)				Interval breast cancer <1 y (n = 324)		Interval breast cancer 1-2.5 y (n = 726)		
	Value	Value	OR (95% CI)	*P* value^b^	Value	OR (95% CI)	*P* value^b^	Value	OR (95% CI)	*P* value^b^
Tumor size, mean (SD), cm	1.43 (1.23)	1.65 (1.45)	NA	<.001	1.97 (1.61)	NA	<.001	1.50 (1.35)	NA	.18
Stage, No./total No. (%)										
In situ	414/1948 (21.3)	178/1032 (17.3)	1.00	<.001	38/321 (11.8)	1.00	<.001	140/711 (19.7)	1.00	.78
Localized	1187/1948 (60.9)	616/1032 (59.7)	1.21 (0.99-1.48)		180/321 (56.1)	1.65 (1.14-2.39)		436/711 (61.3)	1.09 (0.87-1.36)	
Regional	336/1948 (17.3)	221/1032 (21.4)	1.53 (1.20-1.95)		91/321 (28.4)	2.95 (1.97-4.42)		130/711 (18.3)	1.14 (0.87-1.51)	
Distant	11/1948 (0.6)	17/1032 (1.7)	3.59 (1.65-7.82)		12/321 (3.7)	11.89 (4.92-28.74)		5/711 (0.7)	1.34 (0.46-3.94)	
Unknown or missing	21	18	NA		3	NA		15	NA	
Grade, No./total No. (%)										
Well differentiated	417/1701 (24.5)	210/920 (22.8)	1.00	.66	58/273 (21.3)	1.00	.16	152/647 (23.5)	1.00	.93
Moderately differentiated	715/1701 (42.0)	394/920 (42.8)	1.09 (0.89-1.35)		113/273 (41.4)	1.14 (0.81-1.60)		281/647 (43.4)	1.08 (0.86-1.36)	
Poorly differentiated	431/1701 (25.3)	247/920 (26.9)	1.14 (0.91-1.43)		85/273 (31.1)	1.42 (0.99-2.03)		162/647 (25.0)	1.03 (0.80-1.34)	
Anaplastic	138/1701 (8.1)	69/920 (7.5)	0.99 (0.71-1.39)		17/273 (6.2)	0.89 (0.50-1.57)		52/647 (8.0)	1.03 (0.72-1.50)	
Unknown or not done	268	130	NA		51	NA		79	NA	
Lymph node involvement, No./total No. (%)										
Yes	326/1919 (17.0)	211/1013 (20.8)	1.00	.01	83/306 (27.1)	1.00	<.001	128/707 (18.1)	1.00	.50
No	1593/1919 (83.0)	802/1013 (79.2)	1.29 (1.06-1.56)		223/306 (72.9)	1.82 (1.38-2.40)		579/707 (81.9)	1.08 (0.86-1.35)	
Missing	50	37	NA		18	NA		19	NA	
Molecular type, No./total No. (%)^c^										
ER positive/ERBB2 negative	766/1049 (73.0)	417/592 (70.4)	1.00	.50	116/175 (66.3)	1.00	.12	301/417 (72.2)	1.00	.60
ER positive/ERBB2 positive	145/1049 (13.8)	85/592 (14.4)	1.08 (0.80-1.44)		24/175 (13.7)	1.10 (0.68-1.76)		61/417 (14.6)	1.07 (0.77-1.49)	
ER negative/ERBB2 positive	54/1049 (5.2)	30/592 (5.1)	1.02 (0.64-1.62)		14/175 (8.0)	1.71 (0.92-3.18)		16/417 (3.8)	0.75 (0.43-1.34)	
Triple negative	84/1049 (8.0)	60/592 (10.1)	1.31 (0.92-1.87)		21/175 (12.0)	1.65 (0.99-2.77)		39/417 (9.4)	1.18 (0.79-1.77)	
ER positive/ERBB2 unknown	408	202	NA		67	NA		135	NA	
ER negative/ERBB2 unknown	73	47	NA		17	NA		30	NA	
Others^d^	439	209	NA		65	NA		144	NA	
Histologic type, No./total No. (%)										
Ductal	1566/1966 (79.7)	792/1046 (75.7)	1.00	.03	232/322 (72.1)	1.00	.001	560/724 (77.4)	1.00	.34
Lobular	159/1966 (8.1)	101/1046 (9.7)	1.26 (0.97-1.64)		42/322 (13.0)	1.78 (1.24-2.57)		59/724 (8.2)	1.04 (0.76-1.42)	
Ductal and lobular	227/1966 (11.6)	137/1046 (13.1)	1.19 (0.95-1.50)		41/322 (12.7)	1.22 (0.85-1.75)		96/724 (13.3)	1.18 (0.91-1.53)	
Others	14/1966 (0.7)	16/1046 (1.5)	2.26 (1.10-4.65)		7/322 (2.2)	3.38 (1.35-8.45)		9/724 (1.2)	1.80 (0.77-4.18)	
Unknown or missing	3	4	NA		2	NA		2	NA	

Abbreviations: ER, estrogen receptor; OR, odds ratio; NA, not applicable.

^a^ Odds of being in interval breast cancer group. Interval breast cancers were subdivided by their interscreening period: those diagnosed within 1 year of last screening or between 1 and 2.5 years since last screening.

^b^ The χ^2^ test was used for categorical variables, and the *t* test was used for continuous variables. Missing categories are excluded from statistical analysis.

^c^ Calculated excluding ER positive/ERBB2 unknown, ER negative/ERBB2 unknown, and other categories.

^d^ Borderline, ordered/results not available, unknown/not done, and missing. In data submissions earlier than November 2014, borderline ER/PR was not classified positive.^21^

**Figure 2.**
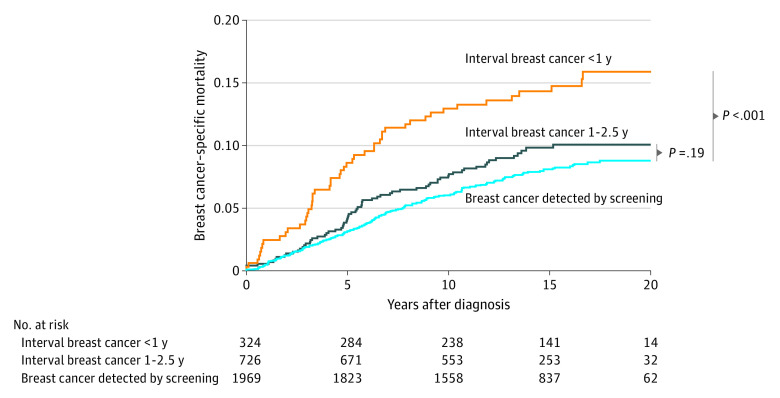
Breast Cancer–Specific Mortality Stratified by Interval Breast Cancer vs Breast Cancer Detected by Screening Overall comparison, *P* < .001 determined by use of the Fine-Gray test; interval breast cancer within 1 year vs breast cancer detected by screening, *P* < .001 determined by use of the Fine-Gray test; and interval breast cancer between 1 and 2.5 years vs breast cancer detected by screening, *P* = .19 determined by use of the Fine-Gray test.

Hazard ratios (HRs) for breast cancer–specific mortality were significantly higher for IBCs diagnosed within 1 year compared with breast cancers detected by screening in unadjusted Fine-Gray competing risks regression models (HR, 1.92; 95% CI, 1.39-2.65) (Figure 2). No breast cancer–specific mortality difference was observed between IBCs diagnosed between 1 and 2.5 years and breast cancers detected by screening (HR, 1.20; 95% CI, 0.90-1.58) (HR, 1.92; 95% CI, 1.39-2.65).

As shown in Table 3, women with IBC diagnosed within 1 year of negative mammogram results were associated with a higher risk of breast cancer mortality compared with those with breast cancers detected by screening after controlling for molecular subtype, histologic type, waist to hip ratio, trial group (HR, 1.69; 95% CI, 1.20-2.37), and either tumor size (HR, 1.46; 95% CI, 1.03-2.08) or lymph node involvement (HR, 1.44; 95% CI, 1.03-2.01). However, the results were no longer statistically significant after further adjusting for both tumor size and lymph node involvement (HR, 1.34; 95% CI, 0.96-1.88) or after controlling for the other common risk factors for breast cancer in model 7 (HR, 1.24; 95% CI, 0.87-1.77). If we add these common risk factors before adding tumor size and lymph node, women with IBC diagnosed within 1 year of negative mammogram results were still associated with a higher risk of breast cancer mortality compared with those with breast cancer detected by screening (HR, 1.64; 95% CI, 1.14-2.34). We replicated our analyses to address lead-time bias and found similar results replacing the survival time from diagnosis date to death or end of follow-up date with date of last mammogram to death or end of follow-up date (eTable 4 in the Supplement). We conducted sensitivity analyses including (1) participants not in the HT trials and (2) only participants in the control groups of both trials and found similar findings to our final model (eTable 2, eTable 3, and eTable 5 in the Supplement).

**Table 3.** Multivariable Fine-Gray Competing Risk Models for Breast Cancer–Specific Mortality: Comparison of Interval Breast Cancers and Breast Cancers Detected by Screening After Controlling for Baseline Health and Tumor Characteristics

Model	Breast cancer type, hazard ratio (95% CI)^a^		
	Detected by screening	Interval breast cancer <1 y	Interval breast cancer 1-2.5 y
1^b^	1 [Reference]	1.81 (1.30-2.51)	1.17 (0.88-1.56)
2^c^	1 [Reference]	1.64 (1.17-2.29)	1.12 (0.83-1.50)
3^d^	1 [Reference]	1.66 (1.18-2.33)	1.17 (0.86-1.60)
4^e^	1 [Reference]	1.69 (1.20-2.37)	1.17 (0.86-1.60)
5^f^	1 [Reference]	1.46 (1.03-2.08)	1.12 (0.82-1.53)
6^g^	1 [Reference]	1.34 (0.96-1.88)	1.13 (0.83-1.55)
7^h^^,^^i^	1 [Reference]	1.24 (0.87-1.77)	1.09 (0.79-1.51)

^a^ This table reports a series of sequential multivariable models in which a new variable is added to each model. Numbers in cells represent the hazard ratios and 95% CIs computed from the Fine-Gray competing risk model. Time is calculated as the time between diagnosis date and end of follow-up. Interval breast cancers were subdivided by their interscreening period: those diagnosed within 1 year of last screening or between 1 and 2.5 years since last screening.

^b^ Fine-Gray competing risk models for breast cancer–specific mortality by breast cancer type adjusting for molecular subtype as the covariate.

^c^ Includes all variables in model 1 with additional adjustment for histologic type.

^d^ Includes all variables in model 2 with additional adjustment for hormone replacement therapy clinical trial group and dietary modification trial group.

^e^ Includes all variables in model 3 with additional adjustment for waist to hip ratio.

^f^ Includes all variables in model 4 with additional adjustment for tumor size.

^g^ Includes all variables in model 5 with additional adjustment for lymph node involvement.

^h^ Includes all variables in model 6 with additional adjustment for other common risk factors for breast cancer (age at diagnosis, race/ethnicity, family history of breast cancer, comorbidity, total number of mammograms before diagnosis, age at menarche, age at first birth, income, educational level, smoking status, alcohol intake, and body mass index).

^i^ If adding the common risk factors in model 7 before adding tumor size and lymph node into the model, the corresponding hazard ratio for interval breast cancer within 1 year is 1.64 (95% CI, 1.14-2.34) and for interval breast cancer between 1 and 2.5 years is 1.16 (95% CI, 0.84-1.59).

## Discussion

In a 19-year breast cancer study among postmenopausal women from the WHI, poor prognostic characteristics (including tumor size, histologic type, and lymph node involvement) were observed specifically for IBCs diagnosed within 1 year of negative screening mammogram results compared with breast cancers detected by screening. Only women with IBCs diagnosed within 1 year of negative screening mammogram results were associated with a significantly higher rate of breast cancer–specific mortality; this lower survival persisted after adjusting for tumor size, molecular type, and histologic type. However, there was no longer a significant difference in breast cancer–specific mortality between women with IBCs detected within 1 year and women with breast cancers detected by screening after further adjustment for lymph node involvement, implicating a potential role for lymph node–mediated metastasis.

The number of IBCs diagnosed in the second year after negative screening mammogram results was more than 2 times higher than that diagnosed in the first year after negative screening mammogram results. However, IBCs diagnosed between 1 and 2.5 years after negative screening mammogram results were not significantly different from breast cancers detected by screening mammograms based on almost all of the clinical and tumor characteristics examined or breast cancer–specific mortality.

Interval breast cancers diagnosed within 1 year of negative mammogram results had a higher proportion of invasive lobular carcinomas than breast cancers detected by screening, consistent with previous reports.^22,23^ Invasive lobular carcinomas account for up to 15% of total invasive breast cancer cases.^24^ Mammography is known to be less sensitive to identifying lobular cancers, in part owing to the fact that lobular tumor cells spread diffusely and as there is lack of mammographic evidence of calcifications, likely owing to loss of E-cadherin calcium-dependent transmembrane protein.^25,26,27,28,29^

Our study corroborates previous research showing that IBC tumors are larger and more likely to be advanced, with more lymph node involvement and a higher proportions of lobular histologic characteristics, than breast cancers detected by screening.^5,6,7,11^ However, previous studies did not compare IBCs by screening interval. The unique findings from our study are that IBCs that emerge within 1 year after negative mammogram results might have distinct tumor characteristics that identify them as at a particularly high risk for metastasis.

Our findings differed from those of previous reports on the associations between patient demographic characteristics and risk for IBC. Previous studies document associations between IBC and family history, parity, and race/ethnicity.^14,30,31^ In contrast, our study found no association with these variables, which may be partly explained by the fact that women in the WHI were postmenopausal and between the ages of 50 and 79 years, whereas other studies included women younger than 50 years.^14,30^

### Strengths and Limitations

This study has some strengths, including (1) a large diverse population with racial/ethnic minority groups, (2) a longitudinal cohort with protocol-mandated screening mammograms during a 10-year period, (3) an extensive follow-up time with recommended screening mammograms, and (4) a robust set of covariates. A unique strength of our study is the ability for stratification by annual or biennial screening intervals. Detailed adjudication of cause of death improved the quality of the data.

This study also has some limitations. In this study, we could not differentiate true IBC from missed breast cancer detected by screening. The systematic review by Houssami and Hunter^12^ synthesized 16 studies and concluded that most IBCs were true IBCs (range, 40%-77%) and estimated that only around 20% to 25% of IBCs were missed during mammographic review. Only a few population-based studies have been able to distinguish true IBC from missed breast cancers, and these studies were conducted in countries with smaller populations and with availability of universal health care.^8,10,32^ It is likely that our sample included some missed breast cancers or false negatives, but these missed cancers likely do not explain the contrast between IBCs diagnosed within 1 year and those diagnosed between 1 and 2.5 years after negative mammogram results. In previous studies, missed cancers had a larger tumor size and lymph node involvement than true IBCs.^25,26,27^ An assessment of missed cancers as IBC is that the cancers diagnosed further in time since the last screening would be later-stage tumors. We did not find this trend and, in fact, observed inverted trends: IBCs diagnosed closer in time to the last mammogram were more likely later-stage tumors (larger and with more lymph node involvement) compared with IBCs diagnosed more distant in time since the last mammogram. These data argue against misdiagnosis being a major confounder of our findings; however, we cannot rule out misclassification due to misdiagnosis in our study.

Another limitation is the lack of data on breast density. High breast density is one of the most important factors associated with IBC regardless of age, and the rate of IBC increases gradually with increasing breast density.^8,10,13,14,28,29^ The primary mechanism by which high breast density is thought to be associated with IBCs is by tumor masking.^33,34^ The idea that increased breast density might be simply an imaging barrier leading to delayed diagnosis may underestimate the role of breast density in IBC. Fibrillar collagen is the dominant tissue component responsible for increased breast density,^35,36^ and fibrillar collagen density, biomechanical properties, and topological architecture may be associated with tumor cell proliferation, invasion, metastasis, and poor outcomes.^33,34,37,38,39^ It remains to be determined whether the ability of collagen to induce aggressive tumor cell phenotypes accounts for poorer prognosis of IBCs.^40,41,42,43^

## Conclusions

Among postmenopausal women from the WHI, IBCs diagnosed within 1 year after negative mammogram results were associated with a larger tumor size, more lymph node involvement, a more lobular histologic type, and higher breast cancer–specific mortality than breast cancers detected by screening. The poor prognosis for women with IBCs diagnosed within 1 year after negative mammogram results might not be due to delayed diagnosis but rather to distinct biological characteristics associated with the cancer. For instance, increased lymph node involvement is often seen in IBCs and cannot be accounted for entirely by delayed diagnosis but rather may be due to a unique biology.

There are several clinical implications of our findings. Women who present with breast cancer symptoms at the time of negative screening mammogram results should either be recalled more frequently, have a shorter screening period, or undergo another imaging modality, such as ultrasonography or magnetic resonance imaging. Use of magnetic resonance imaging can lower the rate of IBCs among high-risk populations.^44,45^ Also, the combination of germline genomic testing with mammography may help distinguish indolent breast cancers from aggressive breast cancers detected by screening.^7^ This study adds to a growing body of literature^46,47,48,49^ that argues for the development of novel approaches to detect life-threatening cancers currently missed by mammographic screening.

## Notes

Supplement. **eTable 1.** Unadjusted Fine-Gray Competing Risk Models for Breast Cancer–Specific Mortality **eTable 2.** Comparison of Tumor Characteristics Between Women With a Diagnosis of Breast Cancer Detected by Screening vs Women With a Diagnosis of Interval Breast Cancer Among Participants Not in HT Trial **eTable 3.** Multivariate Fine-Gray Competing Risk Models for Breast Cancer–Specific Mortality: Comparison of Interval Breast Cancers and Breast Cancers Detected by Screening After Controlling for Baseline Health and Tumor Characteristics Among Participants Not in HT Trial **eTable 4.** Multivariate Fine-Gray Competing Risk Models for Breast Cancer–Specific Mortality: Comparison of Interval Breast Cancers and Breast Cancers Detected by Screening After Controlling for Baseline Health and Tumor Characteristics: Replacing the Survival Time From “Diagnosis Date to Death/End-of-Follow-up Date” With “Date of Last Mammogram to Death/End-of-Follow-up Date” **eTable 5.** Multivariate Fine-Gray Competing Risk Models for Breast Cancer–Specific Mortality: Comparison of Interval Breast Cancers and Breast Cancers Detected by Screening After Controlling for Baseline Health and Tumor Characteristics Among Participants in the Control Arms **eFigure.** Interval Breast Cancer Definition Flowchart
