# Supplementary material for: Comparison of Mortality Among Participants of Women’s Health Initiative Trials With Screening-Detected Breast Cancers vs Interval Breast Cancers
Source: JAMA Netw Open. 2020 Jun 30;3(6):e207227. doi: 10.1001/jamanetworkopen.2020.7227 (PMC7327543; doi:10.1001/jamanetworkopen.2020.7227)
Supplement: Supplement. — eTable 1. Unadjusted Fine-Gray Competing Risk Models for Breast Cancer–Specific Mortality eTable 2. Comparison of Tumor Characteristics Between Women With a Diagnosis of Breast Cancer Detected by Screening vs Women With a Diagnosis of Interval Breast Cancer Among Participants Not in HT Trial eTable 3. Multivariate Fine-Gray Competing Risk Models for Breast Cancer–Specific Mortality: Comparison of Interval Breast Cancers and Breast Cancers Detected by Screening After Controlling for Baseline Health and Tumor Characteristics Among Participants Not in HT Trial eTable 4. Multivariate Fine-Gray Competing Risk Models for Breast Cancer–Specific Mortality: Comparison of Interval Breast Cancers and Breast Cancers Detected by Screening After Controlling for Baseline Health and Tumor Characteristics: Replacing the Survival Time From “Diagnosis Date to Death/End-of-Follow-up Date” With “Date of Last Mammogram to Death/End-of-Follow-up Date” eTable 5. Multivariate Fine-Gray Competing Risk Models for Breast Cancer–Specific Mortality: Comparison of Interval Breast Cancers and Breast Cancers Detected by Screening After Controlling for Baseline Health and Tumor Characteristics Among Participants in the Control Arms eFigure. Interval Breast Cancer Definition Flowchart [file jamanetwopen-3-e207227-s001.pdf]

Supplementary Online Content

Irvin VL, Zhang Z, Simon MS, et al. Comparison of mortality among participants of Women's Health Initiative trials with screening-detected breast cancers vs interval breast cancers. *JAMA Netw Open.* 2020;3(6):e207227. doi:10.1001/jamanetworkopen.2020.7227

eTable 1. Unadjusted Fine-Gray Competing Risk Models for Breast Cancer–Specific Mortality
eTable 2. Comparison of Tumor Characteristics Between Women With a Diagnosis of Breast Cancer Detected by Screening vs Women With a Diagnosis of Interval Breast Cancer Among Participants Not in HT Trial
eTable 3. Multivariate Fine-Gray Competing Risk Models for Breast Cancer–Specific Mortality: Comparison of Interval Breast Cancers and Breast Cancers Detected by Screening After Controlling for Baseline Health and Tumor Characteristics Among Participants Not in HT Trial
eTable 4. Multivariate Fine-Gray Competing Risk Models for Breast Cancer–Specific Mortality: Comparison of Interval Breast Cancers and Breast Cancers Detected by Screening After Controlling for Baseline Health and Tumor Characteristics: Replacing the Survival Time From "Diagnosis Date to Death/End-of-Follow-up Date" With "Date of Last Mammogram to Death/End-of-Follow-up Date"
eTable 5. Multivariate Fine-Gray Competing Risk Models for Breast Cancer–Specific Mortality: Comparison of Interval Breast Cancers and Breast Cancers Detected by Screening After Controlling for Baseline Health and Tumor Characteristics Among Participants in the Control Arms
eFigure. Interval Breast Cancer Definition Flowchart

This supplementary material has been provided by the authors to give readers additional information about their work.

© 2020 Irvin VL et al. *JAMA Network Open.*

eTable 1. Unadjusted Fine-Gray Competing Risk Models for Breast Cancer–Specific Mortality

Variable	Hazard Ratio (95% Confidence Interval)	*P* Value
Breast Cancer Type		
Screening-detected	1.00 (reference)	
Interval breast cancer <1 yr	1.92 (1.39-2.65)	<0.001
Interval breast cancer 1-2.5 yrs	1.20 (0.90-1.58)	0.21
Age at diagnosis	0.99 (0.98-1.01)	0.39
Race		
White	1.00 (reference)	
African American	1.54 (1.06-2.23)	0.02
Hispanic	2.51 (1.47-4.29)	<0.001
Asian	0.63 (0.24-1.68)	0.36
Other	1.59 (0.66-3.86)	0.30
Income		
<$10,000	1.00 (reference)	
$10,000 - $19,999	1.28 (0.59-2.74)	0.53
$20,000 - $34,999	1.07 (0.52-2.22)	0.85
$35,000 - $49,999	1.15 (0.55-2.40)	0.71
$50,000 - $74,999	0.84 (0.40-1.78)	0.65
>$75,000	0.94 (0.44-1.99)	0.86
Smoking		
Never smokers	1.00 (reference)	
Current smokers	1.38 (0.87-2.20)	0.17
Ever smokers	1.20 (0.94-1.53)	0.15
Alcohol		
Never drinkers	1.00 (reference)	
<1 drink/month	0.59 (0.36-0.96)	0.03
<1 drink/week	0.72 (0.47-1.10)	0.13
1-<7 drinks/week	0.76 (0.51-1.15)	0.19
≥7 drinks/week	0.82 (0.52-1.32)	0.42
Past drinkers	0.97 (0.64-1.47)	0.90
Number of mammogram scans before diagnosis	0.95 (0.90-1.00)	0.07
Family history		
No	1.00 (reference)	
Yes	0.94 (0.71-1.26)	0.69
Lymph Node		
No	1.00 (reference)	
Yes	4.53 (3.54-5.79)	<0.001
Size	1.03 (1.02-1.03)	<0.001
Stage		
In Situ	1.00 (reference)	
Localized	3.06 (1.69-5.55)	<0.001

© 2020 Irvin VL et al. *JAMA Network Open.*

Regional	11.95 (6.63-21.56)	<0.001
Distant	114.22 (52.53-248.40)	<0.001
BMI (kg/m^2) at baseline		
Normal (<25)	1.00 (reference)	
Overweight (≥25)	1.13 (0.86-1.48)	0.40
Height (cm) at baseline	0.99 (0.97-1.01)	0.25
Waist-to-hip ratio at baseline		
Normal (<0.80)	1.00 (reference)	
Overweight (≥0.80)	1.29 (1.01-1.65)	0.04
HT Arm Status		
Not randomized to HT	1.00 (reference)	
Estrogen-alone intervention	0.95 (0.55-1.63)	0.85
Estrogen-alone control	1.37 (0.91-2.05)	0.13
Estrogen + Progestin Intervention	1.08 (0.77-1.52)	0.64
Estrogen + Progestin control	0.83 (0.54-1.27)	0.39
DM Arm Status		
Not randomized to DM	1.00 (reference)	
Intervention	0.87 (0.63-1.20)	0.41
Control	1.00 (0.75-1.32)	0.98
Ever full-term birth		
No	1.00 (reference)	
Yes	0.65 (0.36-1.20)	0.17
Comorbidity at baseline		
0	1.00 (reference)	
1	0.96 (0.71-1.29)	0.78
2	1.11 (0.71-1.75)	0.64
≥3	1.52 (0.80-2.86)	0.20
Histology		
Ductal	1.00 (reference)	
Lobular	2.09 (1.51-2.89)	<0.001
Ductal + Lobular	1.16 (0.81-1.68)	0.42
Others	3.28 (1.48-7.25)	0.003
Molecular Subtype		
ER+ and/or PR+/HER2-	1.00 (reference)	
ER+ and/or PR+/HER2+	0.85 (0.51-1.42)	0.53
ER- and/or PR+/HER2+	2.60 (1.53-4.40)	<0.001
Triple Negative	2.47 (1.62-3.77)	<0.001
ER+/HER2 unknown	0.92 (0.66-1.28)	0.62
ER-/HER2 unknown	2.11 (1.31-3.39)	0.002
Others	0.85 (0.60-1.20)	0.34

Each row represents a unique unadjusted Fine-Gray Competing Risk model with that one variable and breast-cancer-specific mortality only included in the model. Numbers in cells represent the hazard ratios, 95% confidence intervals and p-values from that unadjusted risk model. Missing categories are not reported in the table. Variables found significantly related with breast-cancer-specific mortality in these unadjusted models were include in the multi-variate models unless specifically stated.

© 2020 Irvin VL et al. *JAMA Network Open.*

eTable 2. Comparison of Tumor Characteristics Between Women With a Diagnosis of Breast Cancer Detected by Screening vs Women With a Diagnosis of Interval Breast Cancer Among Participants Not in HT Trial

Participant Characteristics	Breast cancer detected by screening (N=1,042)	All Interval Breast Cancer (N=841)		*P* Value[1,2]	Interval Breast Cancer <1 year[5] (N=193)		*P* Value[1,2]	Interval Breast Cancer 1-2.5 year[5] (N=648)		*P* Value[1,2]
	Mean (SD)	Mean (SD)	OR [5] (95% CI)		Mean (SD)	OR [5] (95% CI)		Mean (SD)	OR [5] (95% CI)	
Tumor size, cm	1.45 (1.27)	1.59 (1.41)		0.03	1.88 (1.50)		<0.001	1.50 (1.36)		0.41
	n (%[4])	*n* (%[4])	OR [5] (95% CI)		*n* (%[4])	OR [5] (95% CI)		*n* (%[4])	OR [5] (95% CI)	
Stage				0.25			0.003			0.87
In Situ	219 (21.20)	151 (18.30)	1.00		27 (14.06)	1.00		124 (19.59)	1.00	
Localized	622 (60.21)	502 (60.85)	1.17 (0.92-1.49)		111 (57.81)	1.45 (0.93-2.27)		391(61.77)	1.11 (0.86-1.43)	
Regional	185 (17.91)	162 (19.64)	1.27 (0.95-1.71)		49 (25.52)	2.15 (1.29-3.57)		113 (17.85)	1.08 (0.78-1.49)	
Distant	7 (0.68)	10 (1.21)	2.07 (0.77-5.56)		5 (2.60)	5.79 (1.72-19.53)		5 (0.79)	1.26 (0.39-4.06)	
Grade				0.73			0.52			0.83
Well differentiated	234 (25.43)	178 (23.99)	1.00		42 (25.45)	1.00		136 (23.57)	1.00	
Moderately differentiated	380 (41.30)	317 (42.72)	1.10 (0.86-1.40)		69 (41.82)	1.01 (0.67-1.54)		248 (42.98)	1.12 (0.86-1.46)	
Poorly differentiated	232 (25.22)	195 (2638)	1.11 (0.84-1.45)		46 (27.88)	1.11 (0.70-1.74)		149 (25.82)	1.11 (0.82-1.48)	
Anaplastic	74 (8.04)	52 (7.01)	0.92 (0.62-1.39)		8 (4.85)	0.60 (0.27-1.34)		44 (7.63)	1.02 (0.67-1.57)	
Unknown/not done	122	99			28			71		
Lymph Node Involvement				0.37			0.02			0.96
Yes	207 (19.90)	181 (21.57)	1.11 (0.89-1.39)		53 (27.46)	1.52 (1.07-2.16)		128 (19.81)	0.99 (0.78-1.27)	
No	833 (80.10)	658 (78.43)	1.00		140 (72.54)	1.00		518 (80.19)	1.00	
Molecular Type [4]				0.19			0.05			0.39
ER+/HER2-	390 (74.14)	322 (68.95)	1.00		62 (61.39)	1.00		260 (71.04)	1.00	
ER+/HER2+	70 (13.31)	72 (15.42)	1.25 (0.87-1.79)		17 (16.83)	1.53 (0.84-2.77)		55 (15.03)	1.18 (0.80-1.74)	
ER-/HER2+	29 (5.51)	25 (5.35)	1.04 (0.60-1.82)		9 (8.91)	1.95 (0.88-4.32)		16 (4.37)	0.83 (0.44-1.55)	
Triple Negative	37 (7.03)	48 (10.28)	1.57 (1.00-2.47)		13 (12.87)	2.21 (1.11-4.39)		35 (9.56)	1.42 (0.87-2.31)	
ER+/HER2 unknown	224	166			45			121		
ER-/HER2 unknown	45	40			10			30		
Others[3]	247	168			37			131		
Histology Type				0.19			0.06			0.36
Ductal	827 (79.60)	635 (75.69)	1.00		141 (73.06)	1.00		494 (76.47)	1.00	
Lobular	83 (7.99)	84 (10.01)	1.32 (0.96-1.82)		27 (13.99)	1.91 (1.19-3.05)		57 (8.82)	1.15 (0.81-1.64)	
Ductal + Lobular	121 (11.65)	110 (13.11)	1.18 (0.90-1.56)		24 (12.44)	1.16 (0.73-1.87)		86 (13.31)	1.19 (0.88-1.60)	
Others	8 (0.77)	10 (1.19)	1.63 (0.64-4.15)		1 (0.52)	0.73 (0.09-5.91)		9 (1.39)	1.88 (0.72-4.91)	

1. Chi-square tests are used for categorical variables and t-tests are used for continuous variables.

© 2020 Irvin VL et al. *JAMA Network Open.*

2. Missing categories are excluded from statistical analysis.
3. “Others” include “Borderline, Ordered/Results not available, Unknown/Not done, Missing”. *In data submissions earlier than November 2014, borderline ER/PR was not classified with positive (https://seer.cancer.gov/seerstat/databases/ssf/breast-subtype.html).
4. P-values were calculated excluding “ER+/HER2 unknown”, “ER-/HER2 unknown” and “Others” categories.
5. Odds ratios stand for odds of being in the “interval breast cancer” group. Interval breast cancers were sub-divided by their inter-screening period - those diagnosed within 1 year of last screen or 1 – 2.5 years since last screen.

© 2020 Irvin VL et al. *JAMA Network Open.*

eTable 3. Multivariate Fine-Gray Competing Risk Models for Breast Cancer–Specific Mortality: Comparison of Interval Breast Cancers and Breast Cancers Detected by Screening After Controlling for Baseline Health and Tumor Characteristics Among Participants Not in HT Trial

	**Breast Cancer Type [1]**		
Model	Breast cancer detected by screening	Interval breast cancer <1 yr HR (95% CI)	Interval breast cancer 1-2.5 yr HR (95% CI)
Model 1[2]	1.00 (reference)	1.58 (1.00-2.48)	1.27 (0.92-1.77)
Model 2[3]	1.00 (reference)	1.53 (0.98-2.39)	1.21 (0.86-1.70)
Model 3[4]	1.00 (reference)	1.53 (0.98-2.39)	1.20 (0.85-1.70)
Model 4[5]	1.00 (reference)	1.59 (1.02-2.48)	1.21 (0.86-1.70)
Model 5[6]	1.00 (reference)	1.46 (0.93-2.30)	1.18 (0.83-1.66)
Model 6[7]	1.00 (reference)	1.39 (0.90-2.17)	1.21 (0.86-1.70)
Model 7[8]	1.00 (reference)	1.41 (0.90-2.22)	1.17 (0.82-1.67)

1. This table reports a series of sequential multivariate models where a new variable is added to each model. Numbers in cells represent the Hazard Ratios and 95% confidence intervals computed from the Fine-Grey competing risk model. Time is calculated as the time between diagnosis date and end-of follow-up. Interval breast cancers were sub-divided by their inter-screening period - those diagnosed within 1 year of last screen or 1 – 2.5 years since last screen.
2. Model 1: Fine-Gray Competing Risk Models for breast cancer-specific mortality by breast cancer type adjusting for molecular subtype as the covariate
3. Model 2 includes all variables in model 1 with additional adjustment for histology
4. Model 3 includes all variables in model 2 with additional adjustment for dietary modification trial arm
5. Model 4 includes all variables in model 3 with additional adjustment for WHR
6. Model 5 includes all variables in model 4 with additional adjustment for tumor size
7. Model 6 includes all variables in model 5 with additional adjustment for lymph node involvement
8. Model 7 includes all variables in model 6 with additional adjustment for other common risk factors for breast cancer (age at diagnosis, race/ethnicity, family history of breast cancer, comorbidity, total number of mammograms before diagnosis, age at menarche, age at first birth, income, education, smoking status, alcohol intake and BMI)

© 2020 Irvin VL et al. *JAMA Network Open.*

eTable 4. Multivariate Fine-Gray Competing Risk Models for Breast Cancer–Specific Mortality: Comparison of Interval Breast Cancers and Breast Cancers Detected by Screening After Controlling for Baseline Health and Tumor Characteristics: Replacing the Survival Time From "Diagnosis Date to Death/End-of-Follow-up Date" With "Date of Last Mammogram to Death/End-of-Follow-up Date"

	**Breast Cancer Type** [1]		
Model	Breast cancer detected by screening	Interval breast cancer <1 yr<br>HR (95% CI)	Interval breast cancer 1-2.5 yr<br>HR (95% CI)
Model 1[2]	1.00 (reference)	1.80 (1.30-2.50)	1.13 (0.85-1.50)
Model 2[3]	1.00 (reference)	1.63 (1.17-2.27)	1.08 (0.80-1.44)
Model 3[4]	1.00 (reference)	1.65 (1.17-2.31)	1.12 (0.82-1.53)
Model 4[5]	1.00 (reference)	1.68 (1.19-2.36)	1.13 (0.83-1.54)
Model 5[6]	1.00 (reference)	1.45 (1.02-2.06)	1.07 (0.78-1.46)
Model 6[7]	1.00 (reference)	1.10 (0.76-1.60)	1.08 (0.78-1.49)
Model 7[8, 9]	1.00 (reference)	1.00 (0.68-1.48)	1.00 (0.71-1.39)

1. This table reports a series of sequential multivariate models where a new variable is added to each model. Numbers in cells represent the Hazard Ratios and 95% confidence intervals computed from the Fine-Grey competing risk model. Time is calculated as the time between diagnosis date and end-of follow-up. Interval breast cancers were sub-divided by their inter-screening period - those diagnosed within 1 year of last screen or 1 – 2.5 years since last screen.
2. Model 1: Fine-Gray Competing Risk Models for breast cancer-specific mortality by breast cancer type adjusting for molecular subtype as the covariate
3. Model 2 includes all variables in model 1 with additional adjustment for histology
4. Model 3 includes all variables in model 2 with additional adjustment for hormone replacement therapy clinical trial arm and dietary modification trial arm
5. Model 4 includes all variables in model 3 with additional adjustment for WHR
6. Model 5 includes all variables in model 4 with additional adjustment for tumor size
7. Model 6 includes all variables in model 5 with additional adjustment for lymph node involvement
8. Model 7 includes all variables in model 6 with additional adjustment for other common risk factors for breast cancer (age at diagnosis, race/ethnicity, family history of breast cancer, comorbidity, total number of mammograms before diagnosis, age at menarche, age at first birth, income, education, smoking status, alcohol intake and BMI)
9. If adding the common risk factors in Model 7 before adding tumor size and lymph node into the model, the corresponding HR (95%CI)=1.62 (1.13-2.33) for interval breast cancer<1 yr and 1.11 (0.81-1.53) for interval breast cancer 1-2.5 yr.

© 2020 Irvin VL et al. *JAMA Network Open.*

eTable 5. Multivariate Fine-Gray Competing Risk Models for Breast Cancer–Specific Mortality: Comparison of Interval Breast Cancers and Breast Cancers Detected by Screening After Controlling for Baseline Health and Tumor Characteristics Among Participants in the Control Arms

	**Breast Cancer Type [1]**		
Model	Breast cancer detected by screening	Interval breast cancer <1 yr HR (95% CI)	Interval breast cancer 1-2.5 yr HR (95% CI)
Model 1[2]	1.00 (reference)	1.56 (0.98-2.50)	1.20 (0.84-1.72)
Model 2[3]	1.00 (reference)	1.41 (0.88-2.26)	1.08 (0.74-1.59)
Model 3[4]	1.00 (reference)	1.44 (0.89-2.31)	1.10 (0.75-1.61)
Model 4[5]	1.00 (reference)	1.39 (0.86-2.24)	1.08 (0.73-1.61)
Model 5[6]	1.00 (reference)	1.32 (0.84-2.09)	1.16 (0.78-1.72)
Model 6[7]	1.00 (reference)	1.20 (0.75-1.93)	0.98 (0.65-1.48)

1. This table reports a series of sequential multivariate models where a new variable is added to each model. Numbers in cells represent the Hazard Ratios and 95% confidence intervals computed from the Fine-Grey competing risk model. Time is calculated as the time between diagnosis date and end-of follow-up. Interval breast cancers were sub-divided by their inter-screening period - those diagnosed within 1 year of last screen or 1 – 2.5 years since last screen.
2. Model 1: Fine-Gray Competing Risk Models for breast cancer-specific mortality by breast cancer type adjusting for molecular subtype as the covariate
3. Model 2 includes all variables in model 1 with additional adjustment for histology
4. Model 3 includes all variables in model 3 with additional adjustment for WHR
5. Model 4 includes all variables in model 4 with additional adjustment for tumor size
6. Model 5 includes all variables in model 5 with additional adjustment for lymph node involvement
7. Model 6 includes all variables in model 6 with additional adjustment for other common risk factors for breast cancer (age at diagnosis, race/ethnicity, family history of breast cancer, comorbidity, total number of mammograms before diagnosis, age at menarche, age at first birth, income, education, smoking status, alcohol intake and BMI)

© 2020 Irvin VL et al. *JAMA Network Open.*

eFigure. Interval Breast Cancer Definition Flowchart

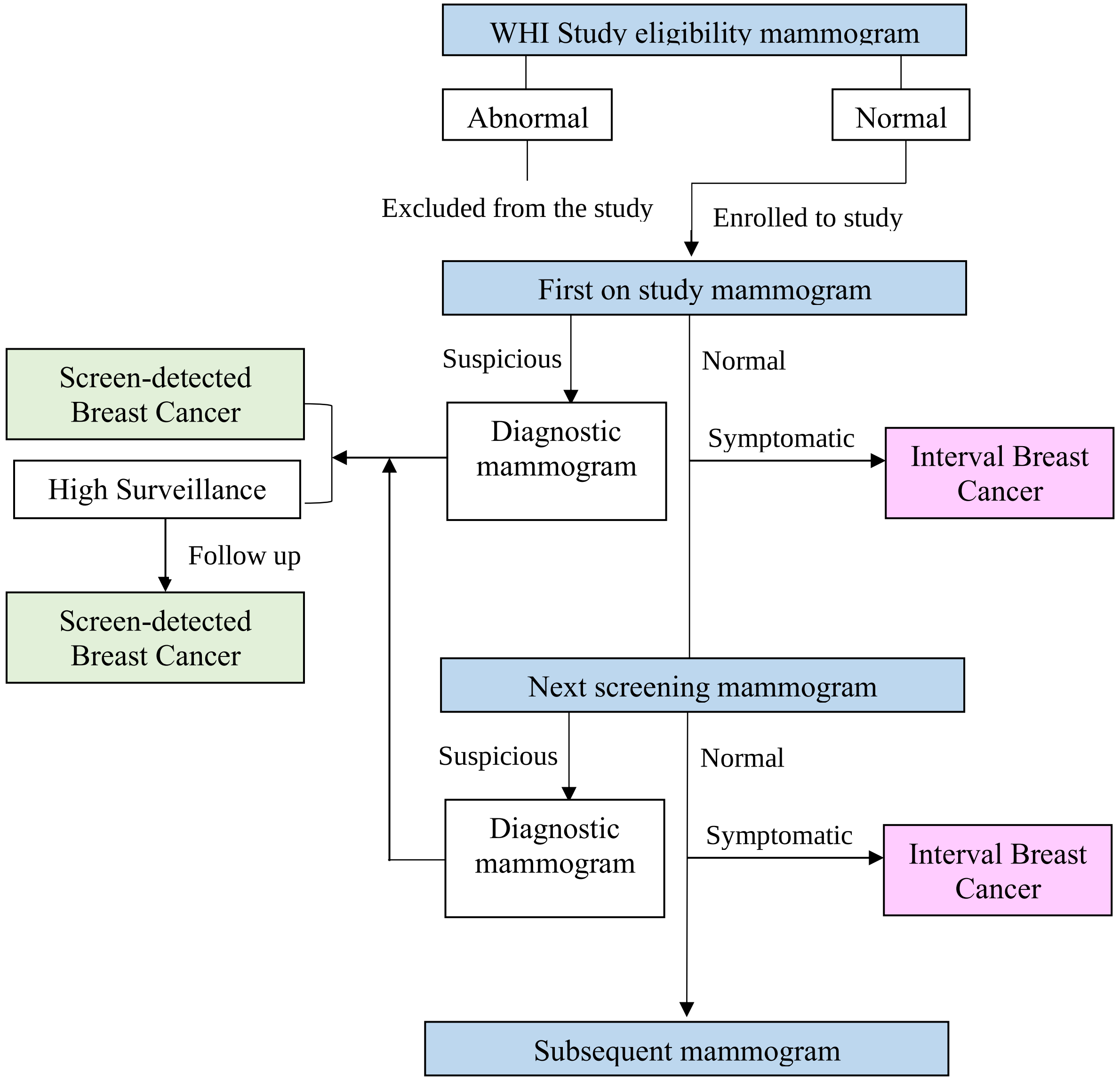

© 2020 Irvin VL et al. *JAMA Network Open.*
